# The Incidence of Double Hypoglossal Canal in Japanese: Evaluation with Multislice Computed Tomography

**DOI:** 10.1371/journal.pone.0118317

**Published:** 2015-02-23

**Authors:** Tomonori Kanda, Tomoki Kiritoshi, Marie Osawa, Keiko Toyoda, Hiroshi Oba, Jun’ichi Kotoku, Kazuhiro Kitajima, Shigeru Furui

**Affiliations:** 1 Department of Radiology, Teikyo University School of Medicine, Tokyo, Japan; 2 Department of Radiological Technology Faculty of Medical Technology, Teikyo University School of Medicine, Tokyo, Japan; 3 Department of Radiology, Kobe University Graduate School of Medicine, Kobe, Japan; University of Liverpool, UNITED KINGDOM

## Abstract

**Background and Purpose:**

Double hypoglossal canal, namely a hypoglossal canal bridging, is a normal variation of the hypoglossal canal. Racial differences in the prevalence of double hypoglossal canal have been reported. We evaluated the prevalence of double hypoglossal canal in a Japanese population with multidetector computed tomography (MDCT).

**Materials and Methods:**

We reviewed five hundred and ninety consecutive patients (mean age, 61 years: range, 15–94 years: 254 men, 336 women) who underwent computed tomographic angiography (CTA) of the brain for a variety of CNS abnormalities. Two radiologists achieved consensus on the canal being single or double, and measured the sizes of single canals on CT images. Kappa statistics was used to test the reliability between the 2 investigators. A logistic regression was used to evaluate the prevalence of double hypoglossal canal and the following factors: sex, age, and laterality. Student’s t-test was used to evaluate the asymmetry of single hypoglossal canal diameters. Statistical significance was accepted at P < 0.05.

**Results:**

Double hypoglossal canal was identified in 16.9% of the patients, and was bilateral in 2.2%. Double hypoglossal canal was significantly more frequent on the left side than right (P = 0.004, odds ratio = 1.79) and in males than females (P = 0.011, odds ratio = 1.67). A larger left or right-sided canal was found in 31.6% and 12.2% of the patients, respectively, following the same side preference as that of double hypoglossal canal. Almost perfect agreement was achieved between the two readers (k = 0.975).

**Conclusions:**

In this Japanese population, the prevalence of a double hypoglossal canal was 16.9%, of which 2.2% were bilateral. Double hypoglossal canal was more frequent in males than females, and on the left side than right.

## Introduction

The hypoglossal canals are paired bony passages that transmit the hypoglossal nerves (CN XII). They run lateral to and slightly anterior to the posterior cranial fossa to the nasopharyngeal carotid space. Besides the hypoglossal nerve, the canal also contains an ample venous plexus, a small variable emissary vein, a branch of the ascending pharyngeal artery, and sometimes a persistent hypoglossal artery [1, 2]. The hypoglossal nerve is formed by 12–16 rootlets that emerge from the preolivary sulcus and usually divide into two trunks that may or may not unite as they traverse the hypoglossal canal. Nonunited trunks may travel separately through a septated hypoglossal canal [3, 4]. Hauser et al. classified the hypoglossal canal into 5 variations using bone specimens as follows: Type 1: No traces of division. Simple canal. Type 2: Traces of division. One osseous spur expressed either marginally at the inner or outer orifice of the canal or inside it. Type 3: Traces of division. Two or more osseous spurs expressed anywhere along the canal. Type 4: Complete osseous hypoglossal bridging expressed in the internal or external portion of the canal. Type 5: Complete osseous hypoglossal bridging extending along the entire canal. In a few recent studies, hypoglossal canal variations were classified into 5 types as above, but most studies have classified bridging as either double (type 4 and 5) or single (type 1, 2, and 3) [5,6].

Multidetector computed tomography (MDCT) scanners easily acquire 1 mm or thinner section thicknesses, allowing isotropic or near-isotropic reconstruction in any plane desired, to easily display the dimensions and morphology of the hypoglossal canal [1, 7]. With magnetic resonance imaging (MRI), high-resolution contrast T1-weighted images (T1WI) with fat suppression can detect the hypoglossal nerve within the enhancing venous plexus [1, 2, 8]. Currently, CT and MRI are the methods of choice for the evaluation of patients with hypoglossal nerve palsy of unknown etiology [1–4,7–12]. Osseous bridging and size asymmetry of the hypoglossal canal are major normal variations. There have been only a few reports on these variations with image analysis, however, and they were based on small numbers of skull specimens [5,6,13–18]. The true prevalence of these variations in the population is not known especially in Japanese. We sought to evaluate the prevalence of hypoglossal canal bridging in a Japanese population using MDCT.

## Materials and Methods

### Ethics Statement

The Teikyo University ethics committee approved this study. Written informed consent was waived because of the retrospective nature of the study. Patient information was anonymized prior to analysis.

### Human subjects

From May 1, 2009 to May 15, 2013, 763 brain CT angiography (CTA) examinations were carried out at our institution. One hundred and twenty-four examinations were additional studies on the same patients and were excluded from this study. Forty-nine scans that did not include thin sections were also excluded. The remaining 590 cases (mean age, 61 years: range, 15–94 years: 254 men, 336 women) were evaluated. The indications for CTA for the 590 patients are shown in Table 1. Of these, contrast-enhanced MRI was carried out in 61 patients, and these images were also evaluated.

**Table 1:** Indications for CT angiography of 590 patients.

	number
cerebral aneurysm	353
brain tumor	98
moyamoya disease	5
arterial stenosis	38
brain infarction	55
arterial dissection	21
venous thrombosis	6
cerebral arteriovenous malformation	7
arteriovenous fistula	2
angiitis	2
cholesteatoma	1
brain abscess	1
infective endocarditis	1

### Imaging protocols

CTA was performed using a 64-detector-row CT system (Aquilion 64; Toshiba Medical Systems, Ohtawara, Japan) with the following parameters: 64 × 0.5 mm detector collimation, reconstructed as axial sections with a thickness of 0.5 mm, slice interval of 0.3 mm, field of view 22 cm, 0.75 s/gantry rotation, 120 kVp, 350 mA and 0.64 beam pitch. Each subject was first examined without contrast material, followed by an injection of iopamidol (Iopamiron 370, Bayer Health Village, Osaka, Japan) with a power injector. The injection dose was 70 mL and the injection rate was 4 mL/s. No saline chaser was administered. A bolus-tracking program was used and the scan delays were set at 5 s after the trigger.

MRI examination was performed using one of two 3 T imaging units (Signa HDxt; GE Healthcare, Milwaukee WI, USA) with an eight-channel head array coil. Axial 3D fat saturation T1WI were acquired after the administration of 0.1 mmol/kg gadobenate dimeglumine. The sequence parameters were repetition time/echo time 6.4/2.1 ms, slice thickness 1.4 mm, slice spacing 0.7 mm, field of view 22 cm, matrix size 352 × 256, flip angle 13°, number of excitations (NEX) 1, and bandwidth 41.67 kHz.

### Image analysis

**Fig 1:**
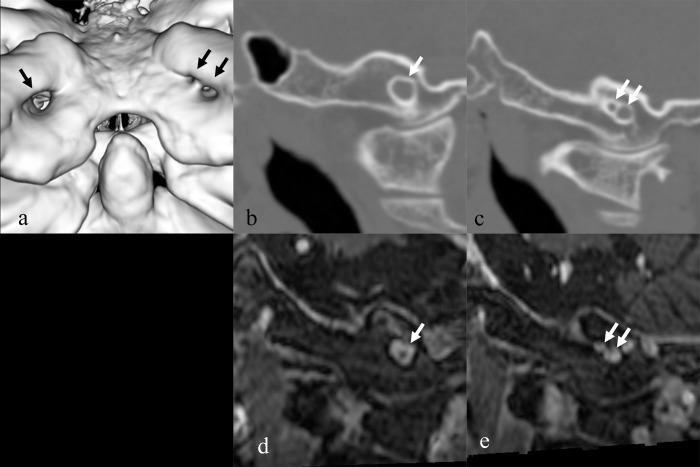
46-year-old female with meningioma had left Double hypoglossal canal. (a) 3D CT image (b) Oblique sagittal reconstructed CT image showed the right hypoglossal canal. (c) Oblique sagittal reconstructed CT image showed left double hypoglossal canal. (d) Low signal intensity of the right hypoglossal nerve within an enhancing venous plexus was seen on oblique sagittal reconstructed image of contrast enhanced and fat saturation T1WI. (e) Low signal intensity of left hypoglossal nerve within a duplicated enhancing venous plexus was seen on oblique sagittal reconstructed image of contrast enhanced and fat saturation T1WI.

The hypoglossal canal was defined as an occipital bone canal extending from the posterior cranial fossa to the nasopharyngeal carotid space. If bone separation existed in the canal on CT, it was defined as ‘double hypoglossal canal’. This definition was the same as Hauser’s definition of hypoglossal canal variation types 4 and 5 [5,6]. Two radiologists (T. K., T. K. with 11 and 3 years of experience, respectively) independently reviewed the non-enhanced and enhanced axial CT results and judged whether double hypoglossal canal was present or not at a window width of 3000 and window level of 500. If discrepancies existed between the two readers, a consensus was achieved by discussion after both reading sessions were completed. The same two radiologists also determined the maximum short-axis diameter of the single hypoglossal canal by consensus. Size asymmetry was defined as a left-right difference > 1 mm. One example of double hypoglossal canal is shown in Fig. 1.

Enhanced MRI images were used to exclude the possibility of the emissary vein being mistaken for the hypoglossal canal. Sixty-one patients had undergone contrast-enhanced MRI, and it was determined whether the hypoglossal nerve was in the canal, as represented by a linear filling defect within the venous plexus of the canal [2].

### Statistical analysis

Kappa statistics was used to test the reliability between the 2 investigators. κ was evaluated as follows: 0–0.20 as slight, 0.21–0.40 as fair, 0.41–0.60 as moderate, 0.61–0.80 as substantial, and 0.81–1 as almost perfect agreement.

A logistic regression was used to evaluate the prevalence of double hypoglossal canal and the following factors: sex, age, and laterality. Student’s *t* test was used to evaluate the sex difference and asymmetry of the single hypoglossal canal diameters. All statistical analyses were performed using software (PASW Statistics, version 21.0; SPSS, Chicago, Ill) and statistical significance was accepted at P < 0.05.

## Results

A total of 113 double hypoglossal canals (in 100 patients) were identified by both readers. Almost perfect agreement was seen between the two readers (k = 0.975). Double hypoglossal canal was found in 16.9% of the subjects, with 14.7% unilateral and 2.2% bilateral. The prevalence of double hypoglossal canal was shown in Table 2, and the logistic regression results were shown in Table 3. Double hypoglossal canal on the left side was significantly more frequent than that on the right (P = 0.004, odds ratio = 1.79) and double hypoglossal canal was significantly more frequent in males than females (P = 0.011, odds ratio = 1.67).

**Table 2:** Prevalence of double hypoglossal canal.

	Male patients (*n* = 254)	Female patients (*n* = 336)	Total (*n* = 590)
Right side	27 (10.6%)**	15 (4.5%)*,**	42 (7.1%)
Left side	35 (13.8%)	36 (10.7%)*	71 (12.0%)
Bilateral	10 (3.9%)	3 (0.9%)	13 (2.2%)

**Table 3:** Factors related to double hypoglossal canal evaluated with logistics regression.

	odds ratio	(95% confidence interval)	P value
**sex**	1.672	(1.127–2.480)	0.011
**age**	0.996	(0.982–1.009)	0.513
**left side**	1.792	(1.199–2.676)	0.004

The sizes of the single hypoglossal canals are shown in Table 4. Left-sided predominant width asymmetry was observed in 31.6% of the patients and right-sided predominant width asymmetry in 12.2%. The left hypoglossal canal in males and females was significantly larger than the right (P < 0.001). Both the right and left hypoglossal canals were significantly larger in males than those in females (P < 0.001).

**Table 4:** Sizes of single hypoglossal canals.

	Male patients (n = 202)	Female patients (n = 288)	Total (n = 490)
Mean size			
Right side	3.27 ± 0.91	2.98 ± 0.91	3.10 ± 0.92
Left side	3.48 ± 0.84	3.21 ± 0.84	3.32 ± 0.90
Asymmetry			
Right > left	26 (12.9%)	34 (11.8%)	60 (12.2%)
Right < left	65 (32.2%)	90 (31.3%)	155 (31.6%)
Right ≈ left	111 (55.0%)	164 (56.9%)	275 (56.1%)

* The left hypoglossal canal in males and females was significantly larger than the right (P < 0.001).

** The hypoglossal canals in males were significantly larger than those in females (P < 0.001).

Of 590 patients, 61 underwent MRI and 13 were noted to have double hypoglossal canal. We verified the hypoglossal nerve within all canals in all cases examined by MRI; no emissary veins were misidentified as hypoglossal canals.

## Discussion

The prevalence of double hypoglossal canal was 16.9%, with 14.7% unilateral and 2.2% bilateral. A sex difference was found in the prevalence of double hypoglossal canal, which was more frequent in male than in female patients (odds ratio = 1.67). Double hypoglossal canal was more frequent on the left side than on the right (odds ratio = 1.79). Size asymmetry of the hypoglossal canal was common, and the right hypoglossal canal was often smaller than the left. The prevalence of double hypoglossal canal followed the same trend as the size of single hypoglossal canals, i.e., it was more common on the left than on the right.

The hypoglossal canals are divided by connective tissue or bone, because they are generally thought to represent the fusion of three or four formerly separated vertebrae in the fetal period, resulting in basioccipital bone formation. Hauser et al. classified the hypoglossal canal into 5 variations using bone specimens. In a few recent studies, hypoglossal canal variations were classified into 5 types, but most studies have classified bridging as either double (type 4 and 5) or single (type 1, 2, and 3) [5,6]. Hauser’s classification was complicated and evaluated very small bone spurs, making the classification difficult even with MDCT. Our results suggest that a sex difference exists in the prevalence of double hypoglossal canal. A previous bone specimen study was carried out using a relatively small number of specimens and the sex difference in the prevalence of double hypoglossal canal was not statistically significant [17]. The sex difference in the prevalence of a double hypoglossal canal found in this study is novel. It is not clear whether this sex difference is a trait of humans in general or limited to the Japanese population. Different prevalences of double hypoglossal canal, based on bone specimens, have been found in different countries, with a range of 7% to 27% [14,17].

According to Dodo, the prevalence of bony hypoglossal canal bridging was anatomically observed in 16.9% of Japanese with a tendency toward left side predominance of bridge formation [19]. Our results show a similar incidence of double hypoglossal canal and left side predominance.

The prevalence of a hypoglossal canal bridging has been used as an anthropological marker to distinguish racial differences. However, our results suggest that the racial differences in the prevalence of a hypoglossal canal bridging may actually be influenced by the sex ratio of the study population.

Size asymmetry of the hypoglossal canal was common in this study, with the right hypoglossal canal often smaller than the left. The reason for this asymmetry is not clear. Okudera et al. suggested that asymmetry of the jugular bulb could occur because of negative pulse waves from the right cardiac atrium to the right jugular bulb through the straight right internal jugular vein [7, 20]. The jugular foramen runs just outside of the hypoglossal canal, and the space for the occipital bone is restricted by the size of the jugular foramen. Asymmetry in the hypoglossal canal size and the prevalence of double hypoglossal canal may be influenced by the asymmetry of the jugular foramen.

Clinically, it is important to evaluate hypoglossal canal bridging to help understand various pathologic processes, such as dural AVF. The venous plexus and emissary vein enter the hypoglossal canal, which plays an important role in the venous circulation. It connects to the anterior condylar confluence and suboccipital cavernous sinus, resulting in widespread antegrade and retrograde drainage in cases with dural AVF. Bony change with dural AVF was reported in this region [21]. Hypoglossal neurinoma, as another disease, shows enlargement of hypoglossal canal [10].

There were some limitations in our study. First, we did not have autopsy results for these cases and there is no proof whether our determination of the status of the hypoglossal canal was true or not. Second, CT has only 0.42 mm/pixel resolution. If the hypoglossal canal was smaller than this, we may have missed the smaller canals and underestimated the prevalence of hypoglossal canal bridging. Third, we could not evaluate the size of the jugular vein with arterial phase contrast-enhanced CT, and so it was difficult to correlate the jugular vein and hypoglossal canal. Fourth, we evaluated only the bridging hypoglossal canals divided by a bony septum. We may have overlooked hypoglossal canals divided by a soft tissue septum or thin bony septum. The double hypoglossal canal may not be equivalent to hypoglossal canal bridging. Moreover, females have smaller hypoglossal canals and this size scaling was likely to be reflected in the thickness of the bridge. Consequently, the sexual difference may be overestimated simply because the smaller female bridges were more difficult to detect than in the males on CT. Finally, some emissary veins may have been mistaken for hypoglossal canal bridging. Although 13 cases of hypoglossal canal bridging were identified using MRI, 88 cases were evaluated by only CT. The emissary vein links the posterior fossa to the hypoglossal canal and usually runs obliquely in the occipital bone. CT can distinguish an emissary vein from the hypoglossal canal. But some emissary veins may be mistaken for a double hypoglossal canal and the prevalence of double hypoglossal canal may be overestimated.

### Conclusions

Double hypoglossal canal was identified in 16.9% of patients, 2.2% of which were bilateral in Japanese. Left greater than right size asymmetry in hypoglossal canals was common. Double hypoglossal canal is more frequent in males than females (odds ratio = 1.67), and is more frequent on the left side (odds ratio = 1.79).

## References

[1] Alves P. Imaging the hypoglossal nerve. Eur J Radiol. 2010;74: 368–377. 10.1016/j.ejrad.2009.08.028 20347541

[2] Voyvodic F, Whyte A, Slavotinek J. The hypoglossal canal: normal MR enhancement pattern. AJNR Am J Neuroradiol. 1995;16: 1707–1710. 7502978 PMC8337779

[3] Yousry I, Moriggl B, Schmid UD, Wiesman M, Fesl G, Brückmann H, et al Detailed anatomy of the intracranial segment of the hypoglossal nerve: neurovascular relationships and landmarks on magnetic resonance imaging sequences. J Neurosurg. 2002;96: 1113–1122. 12066914 10.3171/jns.2002.96.6.1113

[4] Tubbs RS, El-Zammar D, Rogers ME, Kelly DR, Lott R, Chua GD, et al The existence of hypoglossal root ganglion cells in adult humans: potential clinical implications. 2009;Surg Radiol Anat. 31: 173–1 10.1007/s00276-008-0422-6 18853085

[5] Hauser G, De Stefano GF. Variations in form of the hypoglossal canal. Am J Phys Anthropol. 1985;67: 7–11. 3904474 10.1002/ajpa.1330670103

[6] Paraskevas GK, Tsitsopoulos PP, Papaziogas B, Kitsoulis P, Spanidou S, Tsitsopoulos P. Osseous variations of the hypoglossal canal area. Med Sci Monit. 2009;15:BR75–83. 19247236

[7] Tanoue S, Kiyosue H, Sagara Y, Hori Y, Okahara M, Kashiwagi J, et al Venous structures at the craniocervical junction: anatomical variations evaluated by multidetector row CT. Br J Radiol. 2010;83: 831–40. 10.1259/bjr/85248833 20647517 PMC3473745

[8] Linn J, Peters F, Moriggl B, Naidich TP, Brückmann H, Yousry I. The jugular foramen: imaging strategy and detailed anatomy at 3T. AJNR Am J Neuroradiol. 2009;30: 34–41. 10.3174/ajnr.A1281 18832666 PMC7051696

[9] Uchino A, Saito N, Okada Y, Kozawa E, Nishi N, Mizukoshi W, et al Persistent hypoglossal artery and its variants diagnosed by CT and MR angiography. Neuroradiology. 2013;55: 17–23. 10.1007/s00234-012-1074-0 22821359

[10] Santarius T, Dakoji S, Afshari FT, Raymond FL, Firth HV, Fernandes HM, et al Isolated hypoglossal schwannoma in a 9-year-old child. J Neurosurg Pediatr. 2012;10: 130–133. 10.3171/2012.3.PEDS11555 22725844

[11] Borges A, Casselman J. Imaging the cranial nerves: Part I: methodology, infectious and inflammatory, traumatic and congenital lesions. Eur Radiol. 2007;17: 2112–2125. 17323090 10.1007/s00330-006-0575-9

[12] Sakushima K, Terae S, Tsuji-Akimoto S, Niino M, Yabe I, Sasaki H. Idiopathic hypoglossal nerve laceration detected by high-resolution three-dimensional constructive interference in steady state magnetic resonance imaging. J Neuroimaging. 2011;21;e177–179. 10.1111/j.1552-6569.2010.00498.x 20572910

[13] Berlis A, Putz R, Schumacher M. Direct and CT measurements of canals and foramina of the skull base. Br J Radiol. 1992;65:653–61. 1393389 10.1259/0007-1285-65-776-653

[14] Zaidi SHH, Gupta R, Usman N. A study of hypoglossal canal in north indian crania. J. Anat. Soc. India. 2011;60: 224–226

[15] Wysocki J, Kobry H, Bubrowski M, Kwiatkowski J, Reymond J, Skarzyńska B. The morphology of the hypoglossal canal and its size in relation to skull capacity in man and other mammal species. Folia Morphol (Warsz). 2004;63: 11–17. 15039894

[16] Paraskevas GK, Tsitsopoulos PP, Papaziogas B, Kitsoulis P, Spanidou S, Tsitsopoulos P, et al Osseous variations of the hypoglossal canal area. Med Sci Monit. 2009;15: 75–83. 19247236

[17] Eroğlu S. Variations in the form of the hypoglossal canal in ancient Anatolian populations: comparison of two recording methods. Homo. 2010;1:33–47 10.1016/j.jchb.2010.01.002 20097339

[18] Hanihara T, Ishida H. Frequency variations of discrete cranial traits in major human populations. III. Hyperostotic variations. J Anat. 2001;199:251–72. 11554504 10.1046/j.1469-7580.2001.19930251.x PMC1468329

[19] Dodo Y. Appearance of bony bridging of the hypoglossal canal during the fetal period. J Anthropol Soc Nippon. 1980;88:229–38.

[20] Okudera T, Huang YP, Ohta T, Yokota A, Nakamura Y, Maehara F, et al Development of posterior fossa dural sinuses, emissary veins, and jugular bulb: morphological and radiologic study. AJNR Am J Neuroradiol 1994;15:1871–1883. 7863937 PMC8334261

[21] Kondo Y, Kiyose H, Hori Y, Kashiwagi J, Sagara Y, Tanoue S, et al Anterior condylar vein dural AVF with intraosseous vascular nidus in the hypoglossal canal: a case report. Journal of Neuroendovascular Therapy 2007;1: 31–35

